# Gold.—Number IV.

**Published:** 1859-06

**Authors:** 


### GOLD.—Number IV.

BY PROF. AUSTIN.

*Parting by Sulphuric acid.*—Silver must be added to the gold, as in the nitric acid process, but in greater proportion; that is, five of silver to one of gold. There is not the same necessity of adhering to accurate proportions, for the process will work well if there is only one-tenth of gold, and may in fact be profitably conducted where there is only one-thousandth of gold. It is also desirable that there should not be more than one-twentieth of copper in the alloy, for only a limited proportion of sulphate of copper will be dissolved in concentrated sulphuric acid. The preliminary process of cupellation will remove nearly or quite all of the copper; a small quantity is an advantage rather than an injury.

The alloy is first granulated by pouring into water and then introduced, with three or four times its weight of *concentrated* sulphuric acid, into a platina or iron retort, and boiled for two or three hours. The object of using this excess of acid is, that the sulphate formed may with certainty be held in solution. When the silver and copper are completely dissolved the gold is allowed to settle, the liquid decanted, and the gold then thoroughly washed, dried and melted into ingots.

There are several other methods of separating gold from ores or alloys. One is by fusion with sulphur; another is by fusion with sulphide of antimony. A third method, oftener practiced than the first two, is termed *cementation.* The alloy is granulated and mixed in alternate layers, with common salt and brick-dust, in porous crucibles, and heated in a wood-fire for thirty or forty hours. The moisture from wood causes the chlorine of the salt to decompose the silver, and then melting together with other portions of salt, it sinks into the brick-dust, leaving fresh portions of silver to be acted upon by the chlorine. None of these three processes, however, are complete in their results: nine or ten per cent. of silver remains, which must be removed by one of the processes previously described.

Before closing the subject of the refining and purifying of gold, it may not be amiss to give a few practical hints for the management of Dentists' scrap.

Gold-scrap may be divided into three classes: 1. filings; 2. clippings of plate; 3. pieces containing solder and platina. All three are frequently mingled, melted and refined together. But it will, we think, be found more advantageous to sort them, and set aside Nos. 1 and 3 until sufficient in quantity to make it worth the trouble of refining. No. 2 needs no other treatment than simply remelting with a small quantity of borax. It is usual to direct the use of the magnet for the purpose of removing iron from gold filings. But this is open to two objections; some of the gold is apt to be entangled among the iron filings which adhere to the magnet and are lost: again, some particles of iron are apt to be left. We prefer a treatment which removes not only all trace of iron, but also any lead, tin, copper or other metal, or any impurity, *mechanically* mingled with the gold. And that is, to boil the filings for a few moments in pure nitric acid, (pure muriatic might be used, but any mixture of the two must of course be avoided,) and then wash them in water. They may then, without further treatment, be added to class No. 2, provided they are the filings from plate: but if, as usually happens, they are mixed with fragments of platina, and with filings from the solder, they may be added to class No. 3: or treated separately. In melting filings, carbonate of potash is thought by some to act better as a flux than borax: either may be used.

Scrap No. 3 is used by many dentists to make solder, also as an alloy for making plate from coin. But being itself of uncertain fineness, solder and plate of definite fineness or proportions can never be made in this way. Every accurate dentist will prefer to make his plate from coin by adding known weights of pure copper and silver, and to make his solder from pure gold. A laboratory may usually be supplied with what pure gold is required (unless much platina work is done) by refining No. 3 scrap. This is perhaps best done by first melting in a crucible, keeping fluid for half hour or an hour (as it may be more or less impure) and adding from time to time a small piece of nitrate of potash (nitre or saltpetre) and some borax, stirring it with a heated rod, made of porcelain, or of iron coated with fire-clay or glass of borax. The nitre is the oxydizing agent, by virtue of the excess of oxygen it contains: the borax fluxes the oxyds, which otherwise would not fuse, and in this way effectually separates them from the gold, silver and platina. The metal should be allowed to cool in the crucible after this first melting, because of the excess of dross and borax which would interfere with pouring an ingot. When cool, the crucible is to be broken, and with a few blows of a hammer, the borax, etc., separated from the button of gold. Then melt a second time with a very small piece of borax, and pour into a warm, well-oiled ingot mould. After this partial refining by fire, the metal is to be rolled or hammered into a thin strip, and treated by one of the two processes before described—the aqua-regia process, if the gold is *over* eighteen carats fine; if *below* that fineness, the nitric acid process.

A draft furnace gives a steady, uniform heat, and is, therefore, preferable to a blast furnace, which is apt to burn out the coal under the crucible, and chill it by direct contact with the cold blast. The best fuel is hard, well-burnt charcoal, broken into pieces about 1½ inches square. If the pieces are much larger, the fire is not sufficiently compact and burns out too rapidly: if much smaller, the draft is intercepted. It is an excellent plan to select a large close-grained piece upon which to set the crucible: it is well also to pack the smaller coal carefully around this center piece and around the crucible, before lighting the fire. Soft coal burns out too rapidly; coke is more durable than charcoal, but requires a stronger draft: anthracite coal requires a still stronger draft, and is only suitable for long-continued processes: bituminous coal is entirely unsuited for this purpose.

The best crucibles for dentists' use are the Hessian. The inside should be rubbed over with pulverized borax, which glazes the interior and tends to prevent loss by absorption of gold into the crucible. To cover the mouth of the crucible, a piece of sheet-iron may be used, or a piece of firm charcoal: but where a long heat is to be kept up, the best cover is a second crucible of the same size, inverted and securely luted to the first, with a hole in the bottom, through which to introduce the metal and the refining agents and fluxes. One advantage of this kind of cover is, that it enables the fire to be well-built around and above the crucible, and so securing a strong and uniformly-distributed heat, at the same time preventing charcoal from falling upon the metal. Charcoal upon the surface of the melted gold, is an advantage or the reverse according to the object of melting. If it is desired to melt the gold with as little change as possible in the proportions of alloy, then a covering of powdered glass or charcoal may be used to prevent loss by oxydation. But if the object of melting is to refine, then the charcoal is a hindrance, and, whenever nitre is used, directly counteracts its action upon the metal: for red-hot charcoal instantly, and with violence, decomposes nitrate of potash. For this reason, before introducing nitre into a crucible, every particle of charcoal should be carefully blown out with the bellows.

The ingot mould may be of iron, or of soapstone, or of charcoal. If of iron, it should be heated to about the temperature of a laundress' iron, and either oiled or coated with lamp smoke: if of soapstone, it should be somewhat warmed and oiled, but the charcoal mould requires no such preparation. As to the relative merits of those three materials, the iron ingot is most convenient, where much melting is done; but the charcoal gives the *toughest* ingot when small quantities are melted. In explanation of this fact, well known to jewelers, we may perhaps venture to allude to the effect upon iron of gradual or sudden cooling. The same melted iron, which, if poured into a mould of charcoal dust makes a soft tough grey metal, will, if poured into an iron mould, become very hard, brittle and white. If poured into a sand mould, the metal will nearly resemble that poured into charcoal. These changes are attributable to the manner of cooling, which modifies the crystallization of the iron and also its relation to the carbon contained in all steel and cast iron. In gold there is no evidence of the existence of carbon, and thus far the cases are not parallel; but we know that brittle specimens of gold shew a decidedly crystalline structure, which may be owing to the alloy entering into its composition. In which case it will be brittle however managed, until its composition is changed by refining: or it may be owing, as we have supposed, to difference in the manner of cooling. Now, although we could not venture upon *a priori* conjectures, in relation to the effect of gradual or sudden cooling upon the metal, based upon analogy with other metals: yet if such effects are found to exist, as to some extent they undoubtedly do in the case of gold, it is fair to show by comparison with other instances, that it is not so altogether strange as might at first seem.

It is well to distinguish clearly between the effect of sudden cooling from the molten state, and the effect of sudden cooling from the temperature of red-heat. This is the more necessary, because of the well-known property of steel. But gold has, in this respect, no analogy whatever with steel. So far from being made harder by sudden cooling, it is, in the opinion of some experienced workers in precious metals, made rather softer, especially if dipped in alcohol. An extreme case of such effect of sudden cooling, is found in certain bronze alloys for making gongs, which are very brittle until toughened by raising to a red-heat and suddenly cooling in water.

The charcoal ingot may be made by sawing a piece of hard close-grained charcoal in half: then rub the two surfaces together until smooth and true: then either cut out the required shape of the mould in one half, or bend a piece of iron wire, a little thicker than the required ingot, into the proper shape, and lay it between the two pieces. Such an ingot mould needs constant renewal, but, with suitable coal at hand, is not at all difficult to make.

Besides nitre, there are several other refining agents occasionally used; cream of tartar (bitartrate of potash)—sal ammoniac (hydrochlorate of ammonia)—corrosive sublimate (bichloride of mercury). The first is either added in small quantity to the gold in the crucible; or made into a strong solution with water, and the gold poured into it. The theory of its action is not clear: yet the fact is certain, that a lot of metal which perversely remains brittle under the usual treatment with nitre, has often been toughened by its use. The other two substances are usually employed for a similar purpose—to toughen gold which remains brittle after fusion with nitre. They are used in smaller quantity, and dropped into the melted metal. They owe their refining virtues to the chlorine which enters into their composition, as nitre does to its oxygen.

